# Purinergic signaling: a common pathway for neural and mesenchymal stem cell maintenance and differentiation

**DOI:** 10.3389/fncel.2015.00211

**Published:** 2015-06-02

**Authors:** Fabio Cavaliere, Claudia Donno, Nadia D’Ambrosi

**Affiliations:** ^1^Department of Neuroscience, Achucarro Basque Center for Neuroscience, CIBERNED and University of Basque Country, LeioaSpain; ^2^Institute of Anatomy and Cell Biology, Università Cattolica del Sacro Cuore, RomeItaly

**Keywords:** purinergic receptors, ATP, adenosine, mesenchymal stem cells, neural stem cells

## Abstract

Extracellular ATP, related nucleotides and adenosine are among the earliest signaling molecules, operating in virtually all tissues and cells. Through their specific receptors, namely purinergic P1 for nucleosides and P2 for nucleotides, they are involved in a wide array of physiological effects ranging from neurotransmission and muscle contraction to endocrine secretion, vasodilation, immune response, and fertility. The purinergic system also participates in the proliferation and differentiation of stem cells from different niches. In particular, both mesenchymal stem cells (MSCs) and neural stem cells are endowed with several purinergic receptors and ecto-nucleotide metabolizing enzymes, and release extracellular purines that mediate autocrine and paracrine growth/proliferation, pro- or anti-apoptotic processes, differentiation-promoting effects and immunomodulatory actions. Here, we discuss the often opposing roles played by ATP and adenosine in adult neurogenesis in both physiological and pathological conditions, as well as in adipogenic and osteogenic MSC differentiation. We also focus on how purinergic ligands produced and released by transplanted stem cells can be regarded as ideal candidates to mediate the crosstalk with resident stem cell niches, promoting cell growth and survival, regulating inflammation and, therefore, contributing to local tissue homeostasis and repair.

## Purinergic Ligands are Ancient and Widespread Mediators of Cell-to-Cell Communication

It is now widely accepted that in adult organisms stem cells contribute to tissue homeostasis and repair through paracrine mechanisms, along with a mere integration into existing tissue architecture (Wang et al., 2014). Trophic factors combined with immunomodulatory molecules often represent the main mechanism responsible for the functional improvements exerted by transplanted stem cells (Uccelli et al., 2008; Leatherman, 2013). Released nucleotides and nucleosides behave as trophic, differentiating, and immunomodulatory molecules in many physiological and pathological events, through autocrine and paracrine mechanisms (Glaser et al., 2012). Phylogenetically, purinergic ligands are considered ancient molecules involved in cell-to-cell communication, and their receptors are expressed by almost every cell type, even in very primitive organisms such as prokaryotes, protozoa, and early plants (Burnstock and Verkhratsky, 2010). Purinergic receptors are also among the first neurotransmitter receptors to be expressed during very early stages of ontogenetic development (Burnstock and Ulrich, 2011). This conserved and widespread use of purinergic ligands for intercellular communication is possibly due to the fact that nucleotides (and ATP in particular) are fundamental constituents of cells, being the most widely used high energy carrier molecules, and because they are the building blocks of nucleic acids. Cells therefore usually contain millimolar concentrations of intracellular ATP that can be discharged into the extracellular space by vesicular exocytosis, concentrative, and equilibrative transporters, connexin/pannexin hemichannels and uncontrolled leakage from injured cells (Lohman et al., 2012).

Once released into the extracellular environment, purinergic ligands behave as signal mediators, activating different subtypes of purinergic receptors. There are four subtypes of adenosine P1 receptors (A1, A2A, A2B, and A3), seven subtypes of nucleotide P2X ligand-gated ion channel receptors (P2X1–7) and eight subtypes of nucleotide P2Y metabotropic receptors (P2Y1, P2Y2, P2Y4, P2Y6, P2Y11, P2Y12, P2Y13, and P2Y14). The P1 and P2Y subtypes are classical seven-transmembrane domain receptors, whose action is mediated through G-proteins and intracellular second messengers, including Ca^2+^, cAMP, and InsP_3_ (Burnstock, 2007).

The effects of ATP and adenosine are usually opposite and the resulting signal cascade activated by extracellular nucleotides and nucleosides in target cells is the combinatorial resultant of their extracellular metabolism, uptake and binding to specific receptors (Volonté and D’Ambrosi, 2009). Ecto-nucleotide metabolizing enzymes (in particular ecto-nucleoside triphosphate phosphohydrolases, and ecto-5′-nucleotidase) are powerful tools to control the effects mediated by extracellular purines, as they switch off the signal induced by ATP on P2 receptors, hydrolyzing it into adenosine, thereby activating P1 receptors.

Because of their widespread presence and the broad array of functions they can mediate, it is not surprising that purinergic receptors are involved in many aspects of stem cell physiology: mesenchymal stem cells (MSCs) and neuronal progenitor cells (NPCs) release and respond to purinergic ligands with altered proliferation, migration, differentiation and apoptosis, and by regulating immune responses associated with their mobilization (Burnstock and Ulrich, 2011). In this review we will analyze how purinergic signaling behaves as a common paracrine pathway that activates MSCs and neural stem cells (NSCs) in both physiological and pathological conditions.

## Dual Role of the Purinergic System in NSCs in Physiological and Pathological Conditions

### Extracellular Purines Modulate Adult Neurogenesis

**FIGURE 1:**
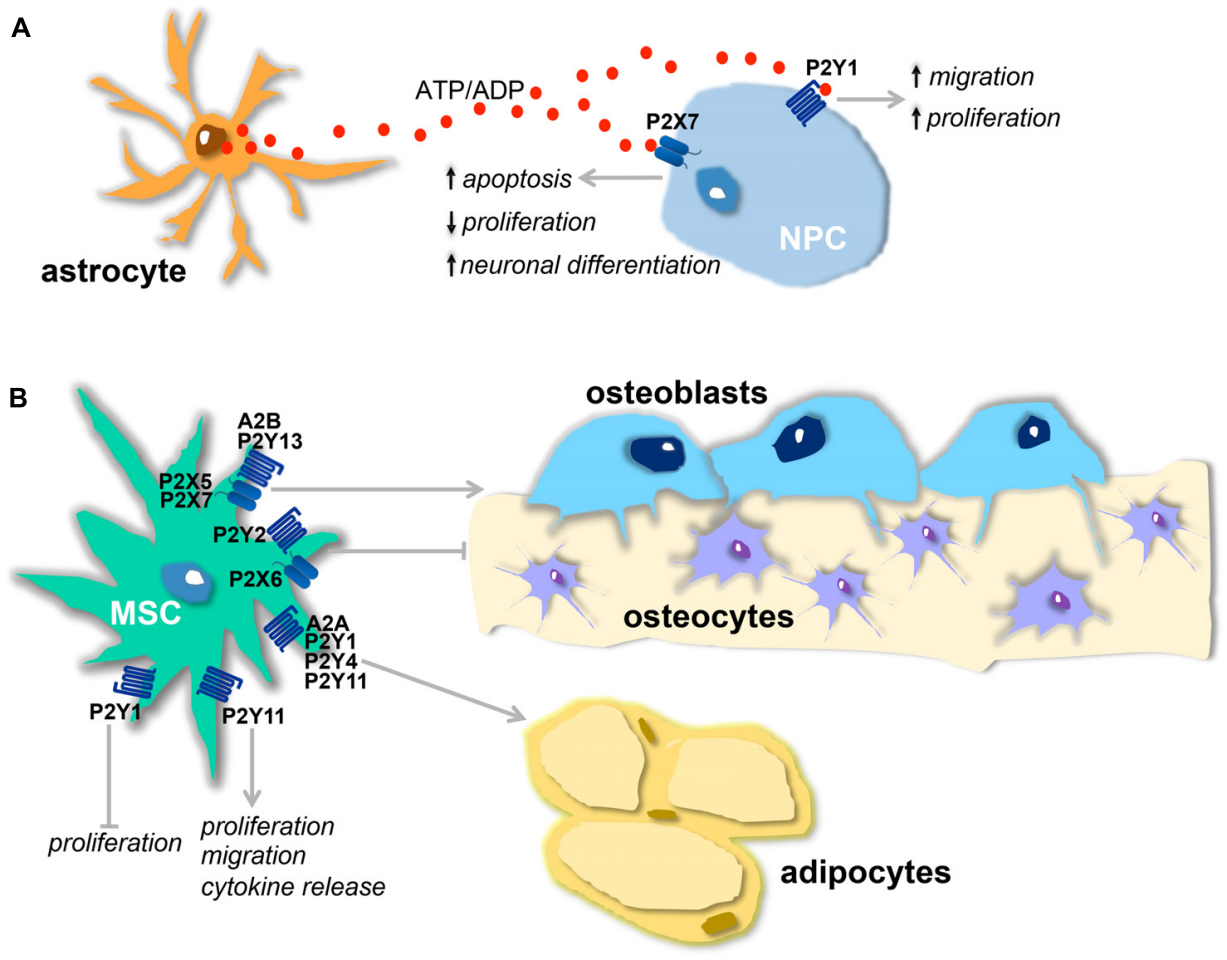
**Physiological effects of purinergic receptors in neural and mesenchymal stem cells (MSCs). (A)** Proposed model of purinergic receptor action on neurogenesis: ATP, released from astrocytes, and ADP, resulting from ATP hydrolysis, stimulate, respectively, P2X7 and P2Y1 receptors present on neural stem cells (NPCs). The activation of P2Y1 receptor leads to increased proliferation and migration and this effect is counterbalanced by P2X7 activation that decreases proliferation, induces neuronal differentiation, and apoptosis. **(B)** Osteogenic and adipogenic actions of purinergic receptors present on MSCs : A2B, P2Y13, P2X5, and P2X7 receptors stimulate osteogenesis, while P2X6 and P2Y2 are inhibitory. A2A, P2Y1, P2Y4 and P2Y11 receptors are adipogenic. P2Y11 receptor also induces migration, cytokine release, and proliferation. Proliferation is inhibited by P2Y1 receptor.

Neural progenitor cells in adult brain express different purinergic receptors. Indeed, mRNAs for P2X4 and P2X7 subtypes, all P2Y receptors except P2Y4 and P2Y11, and all P1 receptors, but A3, have been found in subventricular zone (SVZ)-derived primary neurospheres (Stafford et al., 2007; **Table 1**). Moreover, neural progenitor cells of both SVZ and subgranular zone neurogenic niches highly express the nucleotide-metabolizing enzymes ectonucleoside triphosphate diphosphohydrolase (NTPDase) 2 and the tissue-non-specific alkaline phosphatase (TNAP; Langer et al., 2007). Extracellular nucleotides generated by these enzymes in the SVZ produce a rapid and transient increase in intracellular calcium mainly through the activation of the metabotropic P2Y1 receptor (Mishra et al., 2006). The role of P2Y1 in modulating neurogenesis changes depending on the physiological conditions and the concomitant presence of EGF and FGF. In fact, specific stimulation of this receptor in NPCs increases cell proliferation and migration (Grimm et al., 2010), but only when the growth factor concentration is low or absent (Mishra et al., 2006; Boccazzi et al., 2014; **Table 1**; **Figure 1A**). Conversely, when the growth factor concentration is higher, activation of P2Y1 has an antiproliferative effect (Stafford et al., 2007; **Table 1**). It was recently demonstrated that infusion of ATP in rat SVZ selectively increases the proliferation of type C cells but not of type B or A (Suyama et al., 2012). This effect is counteracted by the selective P2Y1 antagonist 20-deoxy-N6-methyladenosine-30,50-bisphosphate (MRS2179) suggesting a specific role of the P2Y1 receptor in modulating the activity of transit amplifying cells. In line with this, an additional indication of P2Y1 receptor functioning comes from evidence that ATP secreted by astrocytes, even at basal levels, promotes the proliferation of neural progenitor cells through activation of the P2Y1 subunit (Cao et al., 2013; **Figure 1A**).

**Table 1:** Presence and function of purinergic P1 and P2 receptors in neural precursor cells and mesenchymal stem cells.

	Neural precursor cells (NPCs)		Mesenchymal stem cells (MSCs)	
P1/P2	Presence	Effect	Presence	Effect
A1	+	n.d.	+	Lipogenic activity Gharibi et al. (2011)
A2A	+	n.d.	+	Maintainace of osteoblastic differentiation; ↑ adipogenesis Gharibi et al. (2011)
A2B	+	n.d.	+	↑Osteogenesis Ham and Evans (2012)
A3	n.d.	n.d.	+	n.d.
P2X1	n.d.	n.d.	+	n.d.
P2X2	n.d.	n.d.	n.d.	n.d.
P2X3	n.d.	n.d.	+	n.d.
P2X4	+	n.d.	+	n.d.
P2X5	n.d.	n.d.	+	↑Osteogenesis Zippel et al. (2012)
P2X6	+	↓ Migration after ischemia Vergni et al. (2009)	+	↓ Osteogenesis Zippel et al. (2012)
P2X7	+	↓ Proliferation; ↑ neuronal differentiation Tsao et al. (2013) ↑Apoptosis Delarasse et al. (2009), Messemer et al. (2013) ↓ Migration after ischemia Vergni et al. (2009) ↑Innate phagocytosis Lovelace et al. (2015)	+	↑Osteogenesis and mineralization Sun et al. (2013), Noronha-Matos et al. (2014)
P2Y1	+	↑Proliferation Mishra et al. (2006), Boccazzi et al. (2014) ↑Migration Grimm et al. (2010) ↓ Proliferation in the presence of high growth factor concentration Stafford et al. (2007)	+	↓ Proliferation Coppi et al. (2007) ↑Adipogenesis Ciciarello et al. (2013)
P2Y2	+	↑Proliferation Mishra et al. (2006) ↓ Migration after ischemia Vergni et al. (2009)	+	↓ Osteogenesis Zippel et al. (2012)
P2Y4	n.d.	n.d.	+	↑Adipogenesis Zippel et al. (2012), Ciciarello et al. (2013)
P2Y6	+	n.d.	+	n.d.
P2Y11	n.d.	n.d.	+	↑Adipogenesis Zippel et al. (2012) ↑Proliferation, migration, cytochine release Fruscione et al. (2011)
P2Y12	+	n.d.	+	n.d.
P2Y13	+	n.d.	+	↑Osteogenesis, ↓ adipogenesis Biver et al. (2013)
P2Y14	+	n.d.	+	n.d.

*+, Presence; n.d., not detected; ↑, stimulation; ↓, inhibition. The presence of purinergic receptors in NSCs was established by Stafford et al. (2007), in MSCs by Ferrari et al. (2011) and Zippel et al. (2012).*

The effect of P2Y1 in stimulating the proliferation of progenitor cells and neurogenesis can be counterbalanced by activation of the P2X7 receptor (**Figure 1A**). This receptor subtype can regulate the homeostasis of the neurogenic niche, limiting excessive neuro- and glio-genesis by inhibiting proliferation and stimulating NPC differentiation (Tsao et al., 2013) and activating apoptotic mechanisms (Delarasse et al., 2009; **Table 1**). The P2X7 receptor expressed on neuroblasts can also contribute to the clearance of apoptotic cells by activating innate phagocytosis during early stages of neurogenesis (Lovelace et al., 2015; **Table 1**).

### Extracellular Purines Affect NSC Response in Pathological Conditions

Massive release of extracellular ATP is one of the hallmarks of neurodegeneration. After a pathological event in the brain, such as ischemia or Parkinson’s disease all CNS cell types activate different purinergic receptors. P2X7, which is expressed mainly in microglia, astrocytes, and neurons, is the principal agent responsible for purinergic-induced excitotoxic cell death (Sperlagh et al., 2006). Activation of P2X7 in pathological conditions in neurons and astrocytes induces the formation of large pores which, together with pannexin channels, allow the passage of cations, the leakage of metabolites of up to 900 Da and further release of ATP. During an insult extracellular ATP can achieve millimolar concentrations in the extracellular space, determining sustained activation of purinergic receptors and an increase in intracellular calcium in target cells. The imbalance of calcium homeostasis in microglia results in the release of different interleukins, triggering a neuroinflammatory reaction (Sperlagh et al., 2006). However, the role of neuroinflammation in modulating neurogenesis during a pathological event is still debated. Inflammatory cytokines have both a positive and a negative effect on neurogenesis (Borsini et al., 2015) and the activation of purinergic receptors on microglia and astrocytes plays a relevant role in modulating their release. For example, microglial P2X7 activated by its specific agonists ATP and benzoyl-ATP during neuronal stress modulates the expression of NOD-like receptor (NLR) P3 inflammasome (Franceschini et al., 2015), sustaining the release of proinflammatory cytokines which, in turn, may contribute to the inhibition of progenitor cell activity. Conversely, the increase in P2X4 expression in astrocytes contributes to CNS remodeling after trauma and further increases synaptogenesis (Franke and Illes, 2006). Brain ischemia is also characterized by the release of inflammatory cytokines. After an ischemic insult the SVZ is able to release factors that can protect against cortical damage (Cavaliere et al., 2006) and the purinergic system can inhibit this function. Indeed, ATP released after brain insult overstimulates P2 receptors expressed in SVZ progenitor cells (mainly P2X6, P2X7, P2Y1, and P2Y2; Stafford et al., 2007; Vergni et al., 2009), inhibiting the migration of neuroblasts to the damaged cortex (**Table 1**). This process is further enhanced by a locally decreased production of the chemoattractant SDf-1alpha and may also be reversed by blocking the activation of microglia (Vergni et al., 2009). In this case, purines, together with other death signals released by damaged cells, counterbalance the response of progenitor cells recruited after damage (Messemer et al., 2013; **Table 1**).

The general assumption is that, during an insult, ATP can act as a detrimental pro-inflammatory signal, whereas adenosine, mainly through A1 and A3 receptors, usually has opposite properties (Fiebich et al., 2014). It is well known that ATP released after brain injury can be hydrolized by NTPDase2, which is highly expressed in the neural progenitor cell membrane (Gampe et al., 2015), and generate adenosine that, together with the adenosine released directly during brain damage, also has a modulatory effect on neurogenesis (Ulrich et al., 2012).

Finally, an important role in the modulation of NSC function following a stressful event is also exerted by orphan G protein-coupled receptors, which can be activated by extracellular nucleotides. This is the case of GPR17, a novel P2Y receptor specifically activated by both uracil nucleotides (UDP, UDP-glucose, and UDP-galactose) and cysteinyl-leukotrienes (cysLTs; Blasius et al., 1998; Ciana et al., 2006). GPR17 is also expressed in neural progenitor cells, mainly oligodendrocyte precursor cells, and acts as a regulatory factor in mediating oligodendrocyte response and neuronal death after brain ischemia (Lecca et al., 2008).

## Purinergic Signaling in MSCs

Mesenchymal stem cells are self-renewing multipotent stem cells with the capacity to differentiate into chondrocytes, osteoblasts, or adipocytes. Numerous studies have shown that many molecules, inorganic compounds, and mechanical agents contribute to their commitment in the different lineages and it is now clear that there is an inverse relationship between their differentiation into osteoblatsts and into adipocytes. This balance is regulated by intersecting signaling pathways that converge on the regulation of two main transcription factors: peroxisome proliferator-activated receptor-γ (PPARγ) and Runt-related transcription factor 2 (Runx2), which are generally regarded as the master regulators of adipogenesis and osteogenesis, respectively (James, 2013).

Purinergic ligands have been widely described as early factors determining MSC fate (Glaser et al., 2012; Scarfi, 2014) but, while the role of the P1 receptors in MSC physiology is fairly clearly defined, the function of P2 receptors is more controversial, possibly because most of the 15 P2 receptor subtypes have been identified on MSCs (Zippel et al., 2012), it is often difficult to separate the effects of ATP from those of adenosine, and their function seems also to be influenced by the source of origin of the cells. To simplify, ATP can be considered both adipogenic and osteogenic, while its degradation product, adenosine, switches off adipogenic differentiation and has a prevalently osteogenic action (Gharibi et al., 2012; Ciciarello et al., 2013).

### P1 Receptors on MSCs are Mostly Osteogenic and Immunomodulatory

Mesenchymal stem cells release adenosine and possess all P1 receptors (Evans et al., 2006), with A2B as the predominant subtype in undifferentiated cells and during osteoblastogenesis (Gharibi et al., 2011). Not only is adenosine released but most of it derives from the hydrolysis of ATP by ectonucleoside triphosphate diphosphohydrolase 1 (CD39) and ecto-5′-nucleotidase (CD73) activities that are abundantly present in the plasma membrane of MSCs (Sattler et al., 2011). Adenosine exerts an osteogenic action (Ham and Evans, 2012) mainly via the A2B receptor (**Table 1**; **Figure 1B**), its effects being canceled on pharmacological inhibition of this receptor subtype (He et al., 2013), and since overexpression of A2B receptors induces the synthesis of osteoblast-related genes (Runx2 and alkaline phosphatase; Gharibi et al., 2011). Consistently with these *in vitro* results, the knockout of CD73 in mice decreases osteoblast differentiation, resulting in osteopenia (Takedachi et al., 2012); A2B-deficient mice show impaired osteogenic differentiation, a mild osteopenic phenotype and impaired fracture physiology (Carroll et al., 2012); finally, loss of equilibrative nucleoside transporter 1 (ENT1) in mice, with consequent inhibition of adenosine reuptake, leads to ectopic calcification of spinal tissues (Warraich et al., 2013). Adenosine formation and activation of A2B receptors has also been strongly implicated in osteogenic differentiation induced by biomaterials containing calcium phosphate moieties (Shih et al., 2014). The A2A subunit has also been implicated in osteogenesis, being involved mainly in the maintenance of osteoblastic differentiation (**Table 1**) and this P1 subunit, together with the A1 receptor subtype, is also found upregulated during adipogenesis, influencing, respectively, differentiation (through upregulation of PPARγ; **Figure 1**) and lipogenic activity (Gharibi et al., 2011; **Table 1**).

The regenerative effects of MSCs largely depend on their capacity to regulate inflammation and tissue homeostasis via the secretion of an array of immunosuppressive factors, cytokines and growth and differentiation factors that may inhibit inflammatory responses and facilitate the proliferation and differentiation of progenitor cells in tissues *in situ*. P1 receptors are also involved in this aspect of MSC physiology following a pathological insult, being implicated in tissue repair and wound healing by stimulating local repair mechanisms and enhancing the accumulation of endothelial progenitor cells (Katebi et al., 2009). Released adenosine usually displays direct anti-inflammatory effects (Hasko and Pacher, 2008) blocking the proliferation of T-lymphocytes mainly through the A2A subtype, and the addition of A2A antagonists or CD39 inhibitors significantly counteracts this effect (Saldanha-Araujo et al., 2011; Sattler et al., 2011; Lee et al., 2014).

### P2 Receptors have Pleiotropic Effects in MSCs

Human MSCs have been reported spontaneously to release ATP (Coppi et al., 2007) which, in a paracrine way, initiates and propagates intracellular Ca^2+^ waves, promoting the activation of transcription factors that are involved in cell differentiation (Kawano et al., 2006). ATP inhibits the proliferation of bone marrow (BM)-MSCs (Coppi et al., 2007) and stimulates their migration (Ferrari et al., 2011) and PPARγ levels through the activation of different P2X and P2Y receptor subunits (Omatsu-Kanbe et al., 2006; Zippel et al., 2012; Ciciarello et al., 2013; **Table 1**; **Figure 1B**). Together with this adipogenic role for extracellular nucleotides, it was recently demonstrated that P2 receptors are also involved in osteogenesis (**Table 1**) and up- or down-regulation of different P2 subtypes was observed in adipogenic and osteogenic differentiation of MSCs derived from adipose tissue and dental follicles (Zippel et al., 2012). In particular, P2Y13-deficient mice exhibit a decreased bone turnover associated with a reduction in the number of both osteoblasts and osteoclasts (Wang et al., 2014) and MSCs derived from these mice undergo a preferential adipogenic differentiation, showing that the P2Y13 receptor physiologically stimulates the differentiation of osteoblasts (**Figure 1B**) and inhibits that of adipocytes (Biver et al., 2013; **Table 1**). P2X7 receptor activation in BM-MSCs from post-menopausal women and following shockwave treatment also promotes osteogenic differentiation and mineralization (Sun et al., 2013; Noronha-Matos et al., 2014; **Table 1**; **Figure 1B**). Finally, it has been demonstrated that activation of P2Y11 receptor by NAD^+^ released from connexin hemichannels increases proliferation, migration, and cytokine release in BM-MSCs, sparing in this case osteogenic and adipogenic differentiation markers (Fruscione et al., 2011; **Table 1**; **Figure 1B**).

## Purinergic Ligands may be Involved in the Crosstalk between NSCs and MSCs

In this review we have described how purinergic signaling is involved in the physiology of NSCs and MSCs, as both cell types produce and respond to nucleotides and nucleosides. Although purinergic receptors can mediate different effects in the two cell niches (**Figure 1**), in both cases purinergic signaling converges in the modulation of the immune response that is at the basis of stem cell recruitment, in particular after a stressful insult. The activation of P1 receptors is mainly immunosuppressive and trophic for stem cells, while the stimulation of P2 receptors is often proinflammatory and can enhance cell death pathways. Purinergic ligands produced and released by transplanted stem cells can behave as ideal candidates in promoting *in situ* cell growth and decreased apoptosis and in regulating inflammation. For example, although at present there is little evidence of transdifferentiation of MSCs into neurons, it is believed that the secretome of transplanted MSCs can empower surrounding cells to facilitate tissue repair also in CNS pathologies such as stroke, Parkinson’s disease, traumatic brain injury, and epilepsy (Kim et al., 2009; Joyce et al., 2010). With regard to epilepsy, a large body of literature demonstrates the supporting role of adenosine as an endogenous anticonvulsant agent involved in anti-epileptic and anti-apoptotic functions, also by promoting neurogenesis (Glaser et al., 2012; Boison, 2013). Although numerous adenosine agonists have been shown to be potent anticonvulsants in a wide array of animal models of epilepsy, they often produce serious systemic adverse events. An alternative strategy under investigation is to transplant MSCs engineered to release high amounts of adenosine in several models of epilepsy, in order to enhance the natural adenosinergic mechanism triggered by seizures. This approach is very attractive as it provides large amounts of adenosine *in loco*, limiting its action to the foci of seizure and it has indeed proved successful, as engineered MSCs produce a local boost of adenosine and trigger anti-epileptic and anti-apoptotic effects (Boison, 2009; Li et al., 2009; Huicong et al., 2013).

In an acute optic nerve injury model it was shown that MSCs exert neuroprotective and anti-inflammatory effects, also through the down-regulation of the P2X7 receptor in retinal ganglion cells (Chen et al., 2013). Conversely, it was recently shown that ATP released from light-depolarized astrocytes promotes the neuronal differentiation of MSCs through the activation of P2X receptors *in vitro* and *in vivo* (Tu et al., 2014). It is evident from these results that purinergic ligands activate shared pathways that can be involved in MSC and NSC crosstalk, thus allowing mesenchymal and neurogenic niches to become closer.

## Conflict of Interest Statement

The authors declare that the research was conducted in the absence of any commercial or financial relationships that could be construed as a potential conflict of interest.
